# Clinical characteristics and the risk factors for the exacerbation of symptoms in patients with inflammatory bowel disease during the COVID-19 pandemic

**DOI:** 10.3389/fmed.2024.1404880

**Published:** 2024-06-05

**Authors:** Juan Wu, Yuanyuan Fang, Bingqing Bai, Yumei Wu, Qiuyuan Liu, Jing Hu, Naizhong Hu, Qiao Mei, Wei Han

**Affiliations:** Department of Gastroenterology, The First Affiliated Hospital of Anhui Medical University, Hefei, China

**Keywords:** coronavirus disease-19 (COVID-19), inflammatory bowel disease (IBD), exacerbation, symptoms, medication discontinuation

## Abstract

**Background:**

In 2023, the severe acute respiratory syndrome coronavirus 2 (SARS-CoV-2) Omicron variant caused a large-scale outbreak of coronavirus disease 2019 (COVID-19) in China. It is not clear the risk factors that lead to the exacerbation of symptoms in patients with inflammatory bowel disease (IBD) after COVID-19 infection. Our study aims to find out the risk factors for the exacerbation of IBD-related symptoms in IBD patients with COVID-19 infection and to provide guidance for the clinical management of IBD.

**Methods:**

This is a retrospective, observational study. The online questionnaire was distributed to conduct a survey to collect demographic, clinical, and IBD related characteristics in IBD patients. Univariate and multivariate regression analyses were conducted to assess the independent effects.

**Results:**

In total, 534 cases of IBD patients were analyzed in our study. Among them, 466 (87.3%) cases diagnosed with COVID-19, 160 (34.3%) cases experienced exacerbation of IBD symptoms, and 84 (18.0%) patients opted for medication discontinuation. Male sex (OR 2.04, 95% CI 1.34–3.49, *p* = 0.001), and the decrease in body mass index (BMI) (OR 0.93, 95% CI 0.87–1.00, *p* = 0.035) were positively correlated with the exacerbation of IBD symptoms. Furthermore, the medication discontinuation (OR 2.60, 95% CI 1.58–4.30, *p <* 0.001) was strongly positively correlated with the exacerbation of IBD symptoms. No significant association was seen between age, comorbidities, smoking, disease activity, vaccination, therapy for COVID-19 and the worsening of IBD symptoms.

**Conclusion:**

This study confirms that the infection rate of COVID-19 in China IBD patients was comparable to the general population. Male sex, the decrease in BMI and medication discontinuation are significant risk factors for the exacerbation of IBD-related symptoms in IBD patients with COVID-19 infection.

## 1 Introduction

Since the outbreak of coronavirus disease 2019 (COVID-19), the global spread of this pandemic has been swift and far-reaching (1, 2). On 11 March 2020, the World Health Organization officially declared the outbreak a pandemic, marking a significant turning point. With the relaxation of the COVID-19 pandemic in China, the country has experienced a large-scale outbreak of the virus in the past year. Although the majority of COVID-19 cases present with mild symptoms, a small proportion of patients may progress to severe pneumonia, necessitating hospitalization and potentially leading to respiratory failure or fatality (3, 4). Existing clinical evidence showed that people of all age groups were susceptible to contracting COVID-19, however elderly adults and people with underlying diseases were more likely to develop severe pneumonia (5). The individuals who have contracted COVID-19 do not possess durable immunity and are susceptible to recurrent infections (6). Thus, the battle against COVID-19 is a protracted one.

Inflammatory bowel disease (IBD), including ulcerative colitis (UC) and Crohn’s disease (CD), is an immune-mediated inflammatory gastrointestinal chronic disease with increasing prevalence in China (7). Patients with IBD require more elaborate management (7, 8). Most IBD patients are treated with immunomodulators, which can increase the risk of infection. With the spread of the COVID-19 pandemic, there is growing concern about the impact of COVID-19 on patients with IBD. Due to the unique nature of IBD treatment and individualized approach to therapy, managing patients with IBD poses significant challenges during this pandemic (9). Current evidence suggests that there is no difference in COVID-19 incidence and primary clinical outcomes (i.e., hospitalization rate, need for respiratory assistance, and death) between patients with IBD and the general population, but the risk factors that affect the exacerbation of IBD symptoms after COVID-19 infection are unknown (10, 11). The aim of this study is to investigate the impact of COVID-19 on symptoms and medication use in patients with IBD, and to analyze the potential risk factors for the exacerbation of symptoms in IBD patients with COVID-19 infection.

## 2 Methods

### 2.1 Study design

This observational and cross-sectional study was conducted at the Department of Gastroenterology, The First Affiliated Hospital of Anhui Medical University (Hefei, China), from January 4, 2023 to April 17, 2023.

The self-reported questionnaire survey was conducted via Wenjuanxing, which is an online survey platform. Questionnaires were distributed to patients attending the First Affiliated Hospital of Anhui Medical University from January 2023 to March 2023. As the most representative IBD center in Anhui Province, we had a sufficient number of patients. Most of the patients (97.2%) had a long history of residence in Anhui province. The participants were requested to document whether they experienced COVID-19 infection, medication discontinuation, and the subjective exacerbation of IBD symptoms subsequent to contracting COVID-19. Medication discontinuation was defined as no medication up to 2 consecutive weeks in all patients except those treated with infliximab (IFX) and other biologics. For patients treated with IFX and other biologics, medication discontinuation refers to delayed medication for more than 1 week in the induction period and more than 2 weeks in the maintenance period (12). Clinical characteristics and the risk factors for the exacerbation of symptoms in patients with IBD after COVID-19 infection were analyzed.

This study was approved by the Ethics Committee of Anhui Medical University (protocol number: 2023-0134). All patients provided the agreement statement that the participation was voluntary on the first page of the questionnaire.

### 2.2 Participants

Inflammatory bowel disease was diagnosed based on the Chinese consensus on diagnosis and treatment of inflammatory bowel disease (Beijing, 2018). COVID-19 diagnosis was based on viral tests (by nucleic acid or rapid antigen tests, irrespective of symptoms). The exclusion criteria were as follows: (1) The patients had difficulty in understanding the questionnaire. (2) The patients who did not undergo any nucleic acid or rapid antigen tests for COVID-19. (3) The IBD unclassified (IBDU) patients who defined as those with colon IBD but unable to distinguish UC or CD.

### 2.3 Data collection

Sociodemographic information of IBD patients mainly included age, gender, weight, height, occupation, place of residence during the pandemic, underlying diseases, and smoking status. IBD-related data included disease duration and disease activity. The COVID-19-related information included COVID-19 diagnosis status, COVID-19 symptoms, medication, and vaccinations. In addition, we also collected the medication regimens for IBD before and after COVID-19 infection and whether medications were discontinued.

### 2.4 Statistical analysis

We summarized the clinical characteristics of the study population using descriptive statistics, followed by unpaired t tests and Mann–Whitney U tests when appropriate. Univariate and multivariable regression analyses were conducted to assess the independent effects of age, gender, comorbidities, smoking, disease activity, COVID-19 vaccine, COVID-19 treatment, BMI, and medication discontinuation on the exacerbation of symptoms in IBD. Statistical analysis were performed using SPSS-20 software. The goodness of fit of all regression models was evaluated using the Hosmer-Lemeshow test, with a *p* value>0.05 indicating a good fit. A significance level of *p* < 0.05 was considered statistically significant. Finally, the results were plotted using GraphPad Prism software.

## 3 Results

### 3.1 Study population

**Figure 1:**
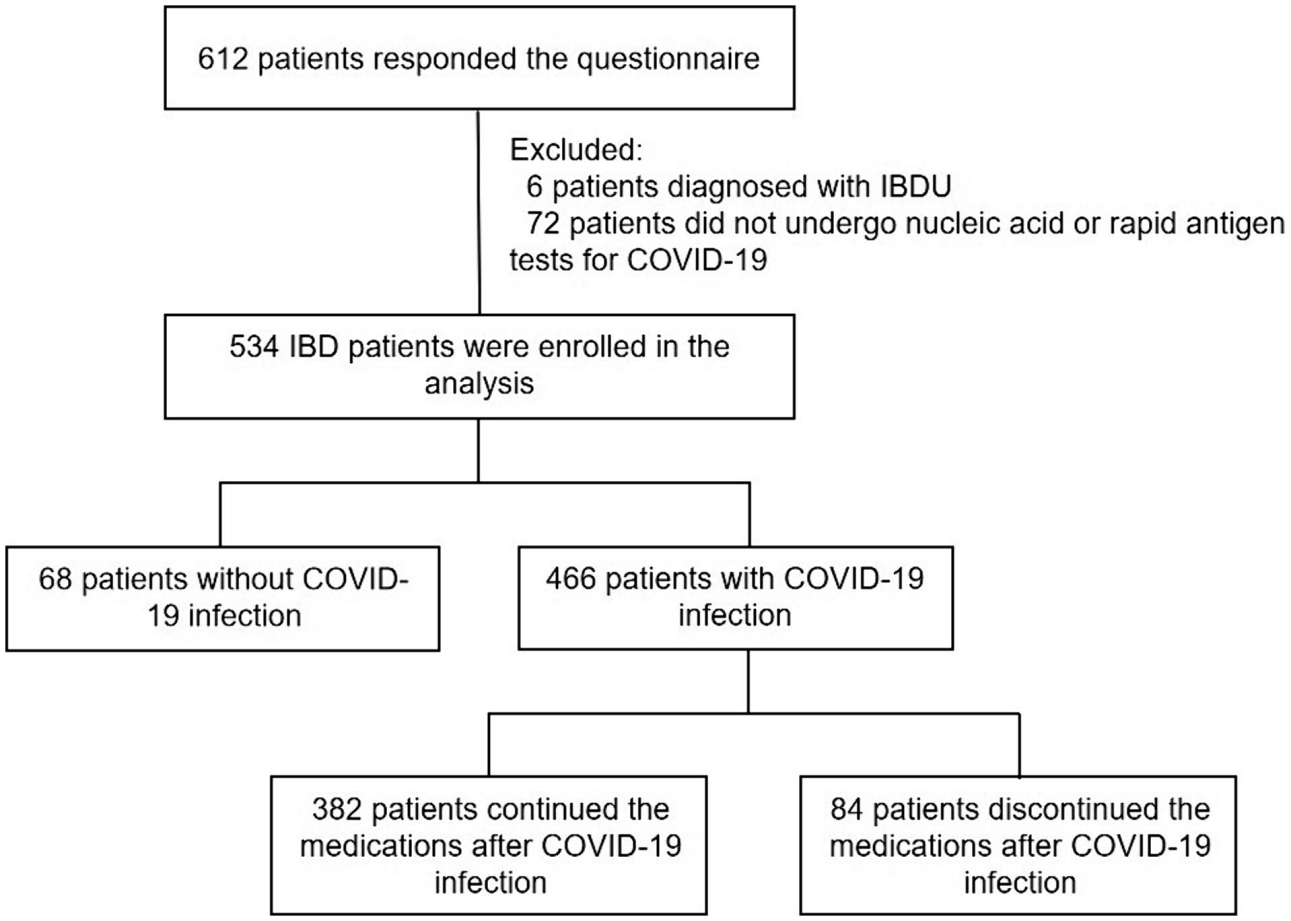
Study population flowchart. COVID-19, coronavirus disease 2019.

A total of 612 IBD patients participated in the survey. 78 patients were excluded due to diagnosed with IBDU (*n* = 6) or did not undergo nucleic acid or rapid antigen tests (*n* = 72). Finally, 534 patients were analyzed, including 466 (87.3%) patients who were diagnosed with COVID-19 infection and 68 (12.7%) patients without COVID-19 infection (Figure 1).

### 3.2 Characteristics of the IBD patients responded to the questionnaire

Demographic, clinical and IBD related characteristics were summarized in Table 1. Among them, there were 424 (79.4%) cases of CD, and 110 (20.6%) cases of UC. 350 (65.5%) patients were male. Also, 395 (74.0%) patients were aged≤40 and 139 (26.0%) aged>40. 33 (6.2%) patients were current smokers. The mean BMI of these patients was 20.5 (18.9–22.8).

**Table 1:** Characteristics of the IBD patients responded to the questionnaire.

	Total (*n* = 534)	Infection (*n* = 466)	Non-infection (*n* = 68)	*p* value
IBD type				0.979
CD	424 (79.4%)	369 (79.2%)	55 (80.9%)	
UC	110 (20.6%)	97 (20.8%)	13 (19.1%)	
Age in years, *n* (%)				0.006
≤40	395 (74.0%)	354 (76.0%)	41 (60.3%)	
>40	139 (26.0%)	112 (24.0%)	27 (39.7%)	
Gender, *n* (%)				0.330
Male	350 (65.5%)	309 (66.3%)	41 (60.3%)	
Female	184 (34.5%)	157 (33.7%)	27 (39.7%)	
BMI, kg/m^2^	20.5 (18.9–22.8)	20.5 (18.8–22.8)	20.5 (19.1–22.4)	0.736
Disease duration, *n* (%)				0.242
≤2	174 (32.6%)	159 (34.1%)	15 (22.1%)	
2–5	219 (41.0%)	183 (39.3%)	36 (52.9%)	
6–10	92 (17.2%)	82 (17.6%)	10 (14.7%)	
>10	49 (9.2%)	42 (9.0%)	7 (10.3%)	
Current smoker, *n* (%)				0.333
No	501 (93.8%)	439 (94.2%)	62 (91.2%)	
Yes	33 (6.2%)	27 (5.8%)	6 (8.8%)	
Comorbidities, *n* (%)				0.970
Without comorbidities	431 (80.7%)	376 (80.6%)	55 (79.4%)	
Cardiovascular disease	2 (0.4%)	2 (0.4%)	0 (0%)	
Hypertension	10 (1.9%)	10 (2.1%)	0 (0%)	
Diabetes	2 (0.4%)	2 (0.4%)	0 (0%)	
Chronic liver disease	16 (3.0%)	16 (3.4%)	0 (0%)	
Respiratory disease	4 (0.7%)	4 (0.9%)	0 (0%)	
Occupation, *n* (%)				0.601
Mental worker	117 (21.9%)	97 (20.8%)	30 (44.1%)	
Manual worker	189 (35.4%)	176 (37.8%)	13 (19.1%)	
Mental-physical worker	16 (3.0%)	13 (2.8%)	3 (4.4%)	
Unoccupied	212 (39.7%)	180 (38.6%)	22 (32.4%)	
Disease activity, *n* (%)				0.058
Active stage	121 (22.7%)	110 (23.6%)	11 (16.2%)	
Inactive stage	291 (54.5%)	255 (54.7%)	36 (52.9%)	
Unknown	122 (22.8%)	101 (21.7%)	21 (30.9%)	

Categorical variables are expressed as *n* (%). Continuous variables are expressed as the medians (Q1–Q3). Comparisons were applied between the infection and non-infection group (Mann–Whitney U test or Fisher’s exact test).

Among these patients, 431 (80.7%) did not have comorbidity disease, and the most common comorbidity were hypertension (1.9%) and chronic liver disease (3.0%). Of all the patients, 121 (22.7%) were in the active stage of IBD, 291 (54.5%) were in remission, and 122 (22.8%) were uncertain.

Comparative analysis between the infection and non-infection groups showed no difference in IBD type, gender, BMI, disease duration, current smoker, comorbidities, occupation, or disease activity. There was a significant difference in age (*p* = 0.006) between infected and non-infected individuals.

### 3.3 Characteristics of IBD patients with COVID-19 infections

The characteristics of patients with COVID-19 infections were summarized in Table 2. A total of 466 patients were infected with COVID-19, including 369 (79.2%) cases of CD and 97 (20.8%) cases of UC. Most patients had multiple symptoms, with fever (85.4%), muscle or joint soreness (58.8%), and fatigue (57.5%) being the most common. However, gastrointestinal symptoms were less common, including diarrhea (15.1%), abdominal pain (4.46%), and hematochezia (0.2%). After being infected with COVID-19, 144 (30.9%) patients did not take any intervention measures, 296 (63.5%) cases chose to self-medicate at home, and 26 (5.6%) cases opted for hospitalization.

**Table 2:** Clinical characteristics of patients with COVID-19 infections.

	All patients (*n* = 466)	Crohn’s disease (*n* = 369)	Ulcerative colitis (*n* = 97)	*p* value
COVID-19-related symptoms, *n* (%)				0.330
Pharyngodynia	143 (30.7%)	106 (28.7%)	37 (38.1%)	
Cough	196 (42%)	152 (41.2%)	44 (45.4%)	
Fever	398 (85.4%)	317 (85.9%)	81 (83.5%)	
Dyspnea	47 (10.1%)	38 (10.3%)	9 (9.3%)	
Fatigue	268 (57.5%)	211 (57.2%)	57 (58.8%)	
Muscle or joint soreness	274 (58.8%)	220 (54.2%)	54 (55.7%)	
Gastrointestinal symptoms	74 (15.9%)	48 (13%)	26 (26.8%)	
No symptoms	16 (3.4%)	11 (3%)	5 (5.2%)	
COVID-19-related interventions, *n* (%)				0.274
Without interventions	144 (30.9%)	117 (31.7%)	27 (27.8%)	
Self-medication at home	296 (63.5%)	232 (62.9%)	64 (66%)	
Hospitalization	26 (5.6%)	20 (5.4%)	6 (6.2%)	
Medical therapy, *n* (%)				0.406
Antivirals	8 (1.7%)	4 (1.1%)	4 (4.1%)	
NSAIDs	280 (60.1%)	222 (60.2%)	58 (59.8%)	
Steroids	2 (0.4%)	2 (0.5%)	0 (0%)	
Cough medicine	96 (20.6%)	71 (19.2%)	25 (26.8%)	
Vaccination, *n* (%)				0.642
Without vaccination	92 (19.7%)	75 (20.3%)	17 (17.5%)	
One dose of vaccine	18 (3.9%)	14 (3.8%)	4 (4.1%)	
Two dose of vaccine	139 (29.8%)	107 (29.0%)	32 (33.0%)	
Three dose of vaccine	211 (45.3%)	170 (46.1%)	41 (42.3%)	
Four dose of vaccine	6 (1.3%)	3 (0.8%)	3 (3.1%)	
Medication discontinuation, *n* (%)				0.474
Yes	84 (18.0%)	65 (17.6%)	19 (19.6%)	
No	382 (82.0%)	304 (82.4%)	78 (80.4%)	

Categorical variables are expressed as *n* (%). Comparisons were applied between the CD and UC group (Fisher’s exact test).

Among the medications used in the treatment of COVID-19, 280 (60.1%) patients took non-steroidal anti-inflammatory drugs (NSAIDs), 96 (20.6%) patients took cough medicine, 8 (1.7%) patients took antivirals, and 2 (0.4%) patients took steroids. Regarding vaccinations, 92 (19.7%) patients did not receive any vaccine, 18 (3.8%) patients received one dose, 139 (29.8%) patients received two doses, 211 (45.2%) patients received three doses, and 6 (1.2%) patients received four doses.

Among the 466 patients infected with COVID-19, 84 (18.0%) individuals discontinued their medication treatment. We further inquired the reasons for medication discontinuation. Among them, 44 (52.4%) patients concerned about drug adverse reactions, which was the primary reason for medication discontinuation. 31 (36.9%) patients were due to transportation inconvenience caused by the COVID-19 pandemic, 20 (23.8%) were limited by inpatient bed shortage, and some other reasons.

There were 306 (65.7%) patients reported no IBD-related symptom exacerbation and 160 (34.3%) patients reported IBD-related symptom exacerbation. 73 (45.6%) patients had IBD-related symptom exacerbation within 1 week after infection, and 37 (23.1%) patients had IBD-related symptom exacerbation within 2 weeks. We found that IBD patients with COVID-19 infection had a greater probability of IBD-related symptom exacerbation within the first 2 weeks.

After COVID-19 infection, 33 patients changed their treatment plans, while 433 patients did not change the treatment plans. In the subset of 33 patients who changed their treatment plans, 23 patients with CD were treated with adalimumab in seven cases, enteral nutrition in nine cases, ustekinumab in four cases, glucocorticoid in two cases, and infliximab in one case; among the 10 patients with UC, seven cases were treated with glucocorticoids, and three cases were treated with traditional Chinese medicine.

### 3.4 Factors related to the exacerbation of IBD-related symptoms

**Figure 2:**
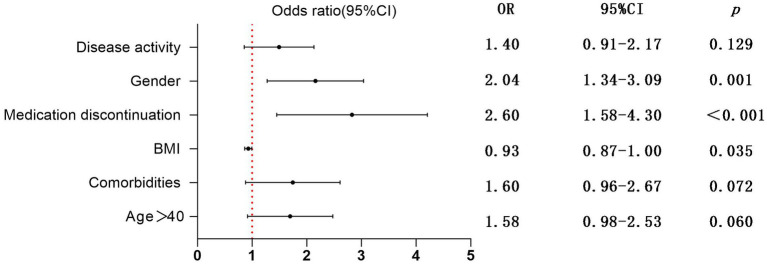
Multivariate regression analysis of risk factors for exacerbating IBD-related symptoms; OR, Odds ratio; CI, Confidence interval.

In this study, we performed univariate and subsequent multivariate regression analyses to explore factors that were more likely to be related to the exacerbation of IBD-related symptoms (Table 3; Figure 2). The univariate analysis showed that male sex (OR 2.22, 95% CI 1.49–3.31, *p < 0.001*), age > 40 (OR 1.70, 95% CI 1.10–2.63, *p = 0.017*), comorbidities (OR 1.81, 95% CI 1.13–2.89, *p = 0.013*), BMI (OR 0.92, 95% CI 0.86–0.98, *p = 0.013*), disease activity (OR 1.63, 95% CI 1.08–2.47, *p = 0.019*), and medication discontinuation (OR 2.85, 95% CI 1.76–4.61, *p < 0.001*) were significantly associated with the exacerbation of IBD-related symptoms. However, in a subsequent multivariate analysis, male sex (OR 2.04, 95% CI 1.34–3.49, *p* = 0.001) and the decrease in BMI (OR 0.93, 95% CI 0.87–1.00, *p* = 0.035) were positively correlated with the exacerbation of IBD symptoms. Furthermore, medication discontinuation (OR 2.60, 95% CI 1.58–4.30, *p* < 0.001) was strongly positively correlated with the exacerbation of IBD-related symptoms.

**Table 3:** Univariate and multivariate regression analysis of risk factors for exacerbating IBD-related symptoms.

Characteristic	Univariate analysis		Multivariate analysis	
	OR(95%CI)	*p* value	OR(95%CI)	*p* value
Gender(man)	2.22 (1.49–3.31)	<0.001	2.04 (1.34–3.09)	0.001
Age(>40)	1.70 (1.10–2.63)	0.017	1.60 (1.00–2.57)	0.052
Comorbidities	1.81 (1.13–2.89)	0.013	1.60 (0.96–2.67)	0.072
BMI	0.92 (0.86–0.98)	0.010	0.93 (0.87–1.00)	0.035
Smoking	0.95 (0.42–2.17)	0.910		
Disease activity	1.63 (1.08–2.47)	0.019	1.40 (0.91–2.17)	0.129
Without vaccination	1.45 (0.91–2.32)	0.117		
Therapy for COVID-19	1.03 (0.68–1.55)	0.906		
Medication discontinuation	2.85 (1.76–4.61)	<0.001	2.60 (1.58–4.30)	<0.001

OR, Odds ratio; CI, Confidence interval.

## 4 Discussion

The COVID-19 pandemic poses the most challenging issue in the era of IBD biology, which has caused a lot of concern and attention among clinical physicians (9, 13). In our study, the infection rate of COVID-19 in IBD patients was 87.3%, comparable to the infection rate among the general population during the same period (13). The data suggests that individuals with IBD are not more susceptible to COVID-19 infection, which is consistent with previous literature reports (14).

We found that patients aged≤40 have significantly higher COVID-19 infection rates than patients aged>40. Comparative analysis between the infection and non-infection groups showed no difference in age in a recent study (11). We consider that young patients had more contact with people for reasons such as work and study, which increased the infection rate of COVID-19. However, elderly patients mostly stayed at home during the COVID-19 pandemic.

Several studies have reported that COVID-19 primarily presented with respiratory symptoms, but can also involve the gastrointestinal tract in some cases (11, 15, 16). However, in our study, gastrointestinal symptoms did not have a higher incidence rate, indicating that the intestines were not the primary target of the SARS-CoV-2 virus. Therefore, patients with IBD do not need to be overly concerned about contracting COVID-19.

It was reported that NSAIDs use had been associated with an increased risk of clinical relapse in IBD patients. However, short-term use of NSAIDs appears to be safe, and the data available suggest that selective COX-2 inhibitors are the safer option. NSAIDs should be avoided as long-term treatment or with high doses, especially in patients with active inflammation (17). In our study, patients with COVID-19 infection usually took NSAIDs in small doses for short periods of time. Meanwhile, we did not find the significant association between therapy for COVID-19 and the exacerbation of IBD symptoms in univariate regression analysis (*p* = 0.906).

The vaccination rate was 80.3% in our patients. Some patients remained skeptical about COVID-19 vaccination. The most common concerns given by patients were the vaccine safety and efficacy (18). Several studies revealed that the COVID-19 vaccine was safe and effective in IBD patients (19–21). Garrido et al. found a high acceptance rate and a good safety profile of SARS-CoV-2 vaccination in IBD patients treated with biologics. Indeed, adverse events were common but overall mild and transitory. These data supported the prioritization and rapid vaccination of these individuals (22). A recent study found that the three vaccination doses were associated with reduced gastrointestinal (GI) symptoms after infection compared with the unvaccinated group (23). There was room for targeted education to improve COVID-19 vaccine uptake in patients with IBD.

Fluctuation of IBD-related symptoms was a concern for most patients and physicians during the infection. Hu et al. (11) reported that over 40% of patients experienced fluctuations in CD-related symptoms after COVID-19 infection, including common GI symptoms and CD-specific manifestations. In our study, 160 (34.3%) patients reported IBD-related symptom exacerbation. On multivariate regression analysis, we found that male sex and low BMI were risk factors for the exacerbation of symptoms in patients with IBD after contracting COVID-19. On the contrary, age, comorbidities, disease activity, vaccination, and therapy for COVID-19 were not significantly related. We also found 84 patients chose to discontinue medication therapy, and multivariate regression analysis showed a strong positive correlation between medication discontinuation and symptom exacerbation. Sahyoun et al. demonstrated that patient factors, including age, sex, race, obesity, and current use of advanced IBD therapies, in the setting of COVID-19 infection were not predictors of post-infectious IBD fares. They observed that the management of COVID-19 had an impact on the temporary interruption of therapy for IBD, but without impact on the exacerbation of IBD symptoms (24).

We further analyzed and revealed that the main reasons for patients discontinuing medication were concerns about drug adverse reactions and transportation inconvenience caused by the COVID-19 pandemic. Most patients experienced symptom exacerbation within 2 weeks after COVID-19 infection, which suggested to clinicians that even if infected with COVID-19, IBD treatment should not be stopped. Furthermore, if there is a delay in medication for some reason, it is also advisable to resume treatment within 2 weeks if possible.

It appears that biologic therapy is not associated with severe COVID-19, providing reassurance that patients can continue biologic therapy (25, 26). Zhang et al. reported that anti-TNF monotherapy, IL12/23 antagonists, and tofacitinib are associated with reduced odds of hospitalization, possibly through abrogation of the cytokine storm induced with severe COVID-19 infection (27). In our study, the majority of the patients did not change their treatment plans due to COVID-19 infection. In the subset of 33 patients who altered their treatment plans, there was an observed increase in the utilization of adalimumab and enteral nutrition among CD patients, as well as an increase in glucocorticoid usage among UC patients. However, due to the small sample size, there may be a significant random element.

Our study has some limitations. Firstly, the study population primarily consisted of individuals from Anhui Province, resulting in a limited sample size. Secondly, the assessment of symptom exacerbation in inflammatory bowel disease heavily relies on patients’ subjective judgment, introducing a certain degree of error. Furthermore, future research should aim to include a more diverse and larger sample size to enhance the robustness of the results.

## Data availability statement

The raw data supporting the conclusions of this article will be made available by the authors, without undue reservation.

## Ethics statement

The studies involving humans were approved by the Ethics Committee of Anhui Medical University. The studies were conducted in accordance with the local legislation and institutional requirements. Written informed consent for participation in this study was provided by the participants’ legal guardians/next of kin. Written informed consent was obtained from the individual(s), and minor(s)’ legal guardian/next of kin, for the publication of any potentially identifiable images or data included in this article.

## Author contributions

JW: Conceptualization, Data curation, Formal Analysis, Methodology, Visualization, Writing – original draft. YF: Conceptualization, Data curation, Formal Analysis, Methodology, Visualization, Writing – original draft. BB: Data curation, Writing – original draft. YW: Data curation, Writing – original draft. QL: Data curation, Writing – original draft. JH: Data curation, Visualization, Writing – original draft. NH: Conceptualization, Methodology, Supervision, Writing – review & editing. QM: Methodology, Project administration, Supervision, Writing – review & editing. WH: Methodology, Project administration, Writing – review & editing.
